# Network embeddedness, digital transformation, and enterprise performance—The moderating effect of top managerial cognition

**DOI:** 10.3389/fpsyg.2023.1098974

**Published:** 2023-02-02

**Authors:** Yue Li, Guo Zhen Fei

**Affiliations:** ^1^YanShan College, Shandong University of Finance and Economics, Jinan, Shandong, China; ^2^School of Business Administration, Shandong University of Finance and Economics, Jinan, Shandong, China

**Keywords:** network embeddedness, digital transformation, enterprise performance, top managerial cognition, questionnaire investigation

## Abstract

Under the new background of the explosive growth of digital economy and the deep integration of real economy, how to improve the performance level through digital transformation has become the key for enterprises to achieve high-quality development. Based on the embeddedness theory and the upper echelons theory, this paper studies the logic and mechanism of network embeddedness affecting enterprise performance, in order to describe the pre-motivations and complete influencing paths of digital transformation affecting enterprise performance, and promote enterprises to achieve high-quality development by means of digital transformation. Taking the middle and senior managers of 239 enterprises as the research objects, this paper applies hierarchical regression, bootstrap and other analysis methods for empirical test, and draws the following conclusions: (1) Relational embeddedness and cognitive embeddedness have a positive impact on digital transformation, while structural embeddedness has no significant impact on digital transformation. (2) Digital transformation has a significant positive impact on enterprise performance. (3) Digital transformation plays a significant mediating role between relational embeddedness, cognitive embeddedness and enterprise performance. (4) In the context of top managerial cognition, cognitive embeddedness can better improve enterprise performance through digital transformation. These results extend the previous literature on digital transformation, proves that digital transformation has a positive effect on enterprise performance. Meanwhile, top managerial cognition is conducive to shaping the dynamic capability of enterprises, and thus plays an important moderating role in the influence path of digital transformation on enterprise performance. This study further affirms the important role of top managerial cognition, which is conducive to enriching enterprises’ digital practices and improving enterprise performance.

## Introduction

With the booming development of mobile Internet and cloud computing, the digital economy is reshaping the world map and becoming a new engine for enterprises to achieve high-quality development. Under the background of the increasingly strong atmosphere of digital transformation, enterprises try to shape the new mode of enterprise control, coordination and collaboration through the transformation and upgrading represented by data technology in order to enhance business value through digital empowerment. Although numerous studies have confirmed the positive impact of digital transformation on enterprise performance (e.g., Dubey et al., 2019; Di and Varriale, 2020; Li L. X., 2022), while other studies believe that digital transformation does not necessarily improve enterprise performance (e.g., Kohtamaki et al., 2020; Ahmadova et al., 2021), because digital transformation brings operational risks to the enterprise, such as data security issues (Corbett, 2018). Moreover, in the process of implementing digital transformation, the enterprise needs through the organization structure optimization, technological upgrading and resource reorganization which are long and costly. In practice, many enterprises will not turn, dare not turn, face a “transition to die, do not transform and wait for death “development dilemma. Based on the contradiction between theory and practice, whether digital transformation can bring performance advantages to enterprises is the primary task of this study.

Digital transformation is not an independent work, and its smooth implementation depends on a large amount of digital technology and information. Most excellent enterprises cannot complete all the innovation links independently, but need to cooperate with multiple companies. For any enterprise in the dynamic and complex network environment, how to integrate the digital knowledge and resources of external partners in the network has become the key to the success of digital transformation. Network embeddedness is defined as the enterprise embedded in the social network jointly formed by its partners (Xie et al., 2021). Enterprises in social network are interdependent and complementary and can share information and obtain resources through connection. Existing studies have proved that the establishment of network connection can enable enterprises to obtain resources from connection relationships, highlighting the important role of enterprise connection (Xie et al., 2021). Therefore, network embeddedness may be an important factor in digital transformation. In addition, the research on the pre-driving factors and the complete influencing path of enterprise digital transformation is still lacking. In order to fill the current gap, the second question is: what is the impact of network embeddedness on digital transformation? Digital transformation is closely related to enterprise dynamic capability. Digital transformation can enhance the enterprise performance by enhancing their dynamic capabilities (Mikalef et al., 2018). The top managerial cognition not only affects the formulation of enterprise strategic behavior (Dutton and Jackson, 1987), but also is the basis for shaping and maintaining dynamic capabilities (Gavetti and Levinthal, 2000). The Accenture China report points out that digital transformation is a “master project,” and its success depends on the strategic support and deployment of the top management of the enterprise. By touching the core of the transformation, digital technology can play an important role in the business, thus highlighting the important role of top managerial cognition. The third question of this study is to explore the role of top managerial cognition in the process of enterprise digital transformation.

Based on this, this paper uses the embeddedness theory and the upper echelons theory to explore the impact of network embeddedness on digital transformation and whether digital transformation can bring performance advantages to enterprises, and builds an integration framework of “network embeddedness-digital transformation-enterprise performance” under the top managerial cognition, so as to describe the complete influence path of digital transformation. It is helpful to deeply understand the mechanism of digital transformation, effectively solve the practical problems of digital transformation, and provide theoretical support for the improvement of enterprise performance in practice.

## Theoretical basis and research hypothesis

The embeddedness theory holds that individuals do not exist independently and their economic behavior is embedded in the social network formed with partners. Enterprises can establish trust relationship, strengthen cooperation and realize resource sharing through connection. Granovetter divided network embeddedness into relational embeddedness and structural embeddedness. Nahapiet enriched this theory by dividing network embeddedness into relational embeddedness, structural embeddedness and cognitive embeddedness (Nahapiet and Ghoshal, 1998), which represents the relationship between individuals or enterprises intimacy, location characteristics and mutual recognition degree. The embeddedness theory provides a better perspective for us to understand the economic behavior between individuals or enterprises. Before the implementation of digital transformation, enterprises must experience a large number of digital technology knowledge sharing, so as to obtain key resources and strengthen organizational learning, and establish the core advantages of digital technology. Therefore, embeddedness can be studied as the pre-motivation for enterprises to implement digital transformation, which helps us to understand the digital technology and knowledge sharing behaviors among enterprises in the process of digital transformation.

Based on their previous work experience, top managerial cognition determines how to understand information in dynamic environments and make strategic decisions based on the understood information (Dutton and Jackson, 1987). As the “personal imprint” of top management, the top managerial cognition is the basis for shaping and maintaining the dynamic capability of an enterprise (Gavetti and Levinthal, 2000). At the same time, when top management receive a large amount of information and cognitive complexity increases, they will reduce their attention to a certain concept, which will help top management overcome cognitive inertia and use the newly acquired information to reconfigure resources, thus reducing organizational inertia. The dynamic capability is closely related to organizational inertia and digital transformation. To be precise, digital transformation itself cannot create additional returns, but it can have a positive impact on enterprise performance by shaping and enhancing dynamic capabilities (Mikalef et al., 2018). Therefore, Warner and Wager (2019) believe that managers should attach importance to shaping dynamic capabilities to play a positive role in digital transformation. In addition, the effectiveness of digital transformation may be hindered by organizational inertia (Mikalef et al., 2021), because the successful implementation of digital transformation also requires changing existing practices within the enterprise and readjust the way of resource allocation (Papadopoulos et al., 2022). In conclusion, in the process of the impact of digital transformation on enterprise performance, top managerial cognition helps to shape and maintain dynamic capabilities and reduce organizational inertia. The above two factors are the key factors to explore the effectiveness of digital transformation. Therefore, top managerial cognition can be introduced into this study, making the theoretical framework of digital transformation more complete.

### Network embeddedness and digital transformation

According to the embeddedness theory, the social network contains rich resources and information, and individuals make decisions and take actions depending on the information resources in the social network. In other words, individual economic behaviors are embedded in the social network. Correspondingly, enterprises are always in a social network of mutual influence and interaction with stakeholders. By establishing close contact with multiple stakeholders, enterprises can obtain needed resources from the network and promote their own development. Nahapiet divides network embeddedness into relational embeddedness, structural embeddedness and cognitive embeddedness (Nahapiet and Ghoshal, 1998), which is used to represent the relationship intimacy, location characteristics and mutual recognition among individuals or enterprises. Digital transformation means that enterprises obtain digital technology resources through the connection with other enterprises in the social network and change the way of collaboration to support business decisions. In this process, the high degree of trust and recognition between enterprises improves the interaction efficiency of information resources, which is conducive to promoting the process of digital transformation. Based on the above analysis, this study proposes the following hypothesis.

*Hypothesis 1*: Network embeddedness has a positive impact on digital transformation.

Relational embeddedness not only refers to the assets created and utilized through relationships (Nahapiet and Ghoshal, 1998), but also emphasizes the degree of trust and respect between individuals. First of all, in social networks, a higher degree of relational embeddedness means mutual trust, enhances the willingness to share knowledge and information, and promotes the realization of knowledge transfer among individuals. In the process of enterprises’ implementation of digital transformation, high trust is conducive to enterprises in the social network to make high resource commitment such as knowledge and technology for the collective, and realize the efficient transfer of digital transformation knowledge among enterprises. Secondly, high trust is conducive to long-term cooperation between enterprises and lower transaction costs (Tiwana, 2008). The close connection between enterprises increases the cost of normative cooperation and violation of network conventions, reduces the speculative behavior in the transaction process (Gonzalez-Brambila et al., 2013), and promotes the gradual implementation of the digital transformation of clusters. Finally, the high degree of trust generated between the actors who interact frequently enhances the willingness of enterprises to act as “intermediaries,” “introduce” other new enterprises, and realize the release of a wider range of digital technology and other information (Tiwana, 2008), thus enhancing the possibility of enterprises obtaining valuable digital technology and other resources. Based on the above analysis, this study proposes the following hypothesis.

*Hypothesis 1a*: Relational embeddedness has a positive impact on digital transformation.

Structural embeddedness can be measured by the index of network centrality and network scale. Network centrality represents the degree to which an enterprise occupies a dominant position in a social network, and network scale represents the number of enterprises in a social network. Network centrality can be analyzed by communication degree, resource allocation potential and independence index. First of all, enterprises with high centrality are in direct contact with other enterprises and occupy a dominant position in the social network. Through direct communication with more enterprises, they are more likely to obtain high-quality resources in the connection process. Secondly, centrally located enterprises have the ability to control information flow and coordinate resources (Cohn and Marriott, 1958). When enterprises in a central position implement digital transformation, they can dispatch high-quality resources in the social network through their own coordination ability to promote digital transformation. Finally, compared with other enterprises, centrally located enterprises can directly contact more enterprises without relying on information intermediaries, which is more independent and efficient (Bavelas, 1950). The information technology and other resources required by central enterprises in the implementation of digital transformation can be obtained in the shortest time without other enterprises acting as intermediaries, which saves time cost and improves the efficiency of information interaction. In addition, from the perspective of network scale, the larger the network scale, the more diversified the structure, and the more channels for enterprises to obtain external resources, which means that they have diversified sources of knowledge (Cummings, 2004) and then absorb more heterogeneous knowledge to provide support for the implementation of digital transformation. Based on the above analysis, this study proposes the following hypothesis.

*Hypothesis 1b*: Structural embeddedness has a positive impact on digital transformation.

Cognitive embeddedness includes the common language and concentrated narrative in the long-term communication process of enterprises in the social network, which reflects the mutual understanding, common vision and goal between enterprises (Nahapiet and Ghoshal, 1998). First of all, shared vision and goals enhance mutual recognition and collective standardization. Enterprises tend to adjust their own behaviors according to other enterprises and groups as the reference frame, so that their behaviors tend to be consistent (Lieberman and Asaba, 2006). The enterprise who takes the lead in promoting the digital transformation has deeply understanding of digital technology, resources, policy and can take advantage of a party or conference to promote digital information flow between the other enterprises. The common values which enterprises have make heterogeneous knowledge be more likely to be accepted and understood by other enterprises in the network, and then make adjustments for enterprises’ actions. Secondly, high cognitive embeddedness reflects the sharing culture formed by long-term communication between enterprises, which has a higher understanding of each other and can reach a consensus on controversial issues in the implementation of digital transformation (Pomegbe et al., 2020), effectively promoting knowledge sharing and joint problem solving between enterprises, forming a closer cooperative relationship and realizing digital transformation. Based on the above analysis, this study proposes the following hypothesis.

*Hypothesis 1c*: Cognitive embeddedness has a positive impact on digital transformation.

### Digital transformation and enterprise performance

At present, there are many researches on the improvement of enterprise performance by digital transformation. Combing through the existing researches, it is found that digital transformation has an impact on enterprise performance through two paths: enterprise cost and capital use efficiency.

Through digital empowerment, enterprises can reduce production costs and transaction costs and improve corporate performance. First of all, in the production process, the application of digital technology is conducive to the enterprise to monitor the running condition of the machine and reduce the time cost of machine maintenance (Moeuf et al., 2018). The data-driven production system avoids the errors caused by human subjective consciousness and realizes the intelligent and controllable production. With the support of digital technology, enterprises can accurately locate user needs to achieve flexible and modular production, which is conducive to improving the ability of assembly line adjustment, strengthening production collaboration and reducing resource loss on the premise of meeting customer needs (Zhang et al., 2022). Secondly, enterprises overcome regional culture and language barriers through digital transformation to reduce the cost of collaboration between enterprises (Goldfarb and Tucker, 2019; Tsou and Chen, 2021). In the enterprise, efficient information management system and communication software are introduced through digital technology to reduce the information acquisition cost of all functional departments and improve the approval efficiency (Liang and Li, 2022). The full flow of information further improves the cooperation level of all functional departments and reduces the communication cost. Finally, digital technology speeds up the frequency of information interaction between enterprises, the government and regulatory authorities, helps enterprises obtain panoramic information, and then avoids bounded rationality and opportunism in decision-making to reduce transaction costs (Sankaranarayanan and Sundararajan, 2010).

Digital transformation is conducive to improving the use efficiency of human capital and material capital, and improving corporate performance. First of all, digital transformation speeds up the automation of decision making, greatly reduces the number of simple jobs, and encourages employees to improve their digital skills and shift to more valuable jobs, highlighting the value of intellectual capital (Trenerry et al., 2021). Existing studies have shown that intellectual capital contributes more to value creation and appreciation than financial capital, which helps enterprises realize human capital appreciation and improve economic profits (Ainunnisa, 2021). Secondly, the platform based on digital technology can not only strengthen the connection between enterprises and users and the supply chain, but also allocate production factors according to the information obtained in real time, so as to help enterprises rationally arrange production plans and greatly reduce material loss and resource waste (Wang et al., 2022). Finally, digital technology realizes the interconnection between enterprises, enables enterprises to capture asset information in a more timely manner, helps enterprises realize the reuse of idle assets, solves the problem that small enterprises cannot afford to purchase high-value machines, and thus improves the efficiency of internal asset use and performance improvement. Based on the above analysis, this study proposes the following hypothesis.

*Hypothesis 2*: Digital transformation has a positive impact on enterprise performance.

### The mediating role of digital transformation in network embeddedness and enterprise performance

Based on the logic of the above assumptions, it can be seen that the higher the degree of enterprise network embeddedness, the higher the degree of mutual trust, recognition and network centrality, the more efficient information resource interaction between enterprises can be guaranteed. Digital transformation is a process with large investment and long pain period, which requires extensive and efficient information resource interaction by enterprises to proceed smoothly. A high degree of network embeddedness promotes digital transformation by ensuring efficient information resource interaction between enterprises. Through the continuous implementation of digital transformation strategy, enterprises can break the organizational boundaries and realize the interconnection of network member enterprises (Borkar et al., 2012). These behaviors can reduce transaction costs (Sankaranarayanan and Sundararajan, 2010), strengthen the link with consumers, and improve the efficiency of capital use by reducing material consumption through flexible production (Zhang et al., 2022). In addition, the implementation of digital transformation strategy helps enterprises benefit from knowledge spillover. The essence of knowledge spillover is the transmission of information, and space affects the flow and transmission of information (Fung, 2006). Digital platform accelerates the knowledge spillover of R&D cooperation between enterprises, and helps backward enterprises to benefit from external knowledge spillover effect through observation and learning (Zhang et al., 2014). Based on the above analysis, this study proposes the following hypothesis.

*Hypothesis 3*: Digital transformation mediates the relationship between network embeddedness and enterprise performance.

### The moderating effect of top managerial cognition

The sustainability of investment and the unpredictability of results make digital transformation become a major strategic decision of enterprises. The purpose of digital transformation is to digitally empower all business processes and improve performance. However, if only digital technology is used to deal with business operations, it will not improve enterprise performance. Only by elevating the digital transformation to the strategic level, guiding the organization to implement the strategic transformation from top to bottom, and shaping the organizational capacity in line with the strategic decision, can enterprises maximize the positive role of digital transformation and improve the performance level. As the “personal imprint” of senior executives, the top managerial cognition not only influences their interpretation of the internal and external environment of the enterprise, shapes their decision preferences, and influences the formulation of corporate strategic behaviors (Dutton and Jackson, 1987), but also serves as the basis for shaping and maintaining the dynamic capability of the enterprise (Gavetti and Levinthal, 2000). Therefore, the top managerial cognition can be used as a situational variable to determine whether digital transformation can better improve enterprise performance. That is, with different levels of top managerial cognition, the degree to which digital transformation can improve enterprise performance is different.

First of all, the higher the level of top managerial cognition, the easier it is to form strategic decisions dominated by digital transformation within the enterprise. In the social network, the frequent interaction of digital technology and information improves the attention of top managers to digital technology and other information in the dynamic environment, integrates the digital knowledge received and learned from the external environment into the cognitive knowledge structure, increases the complexity of top managerial cognition (Nadkarni and Narayanan, 2007), and reduces the cognitive concentration of specific concepts. It is helpful for top managers to overcome cognitive inertia (Carley and Palmouist, 1992), cultivate diversified cognitive perspectives, and incorporate digital transformation into corporate strategic decisions by deepening their understanding of digital information. Secondly, the top managerial cognition can help enterprises to form dynamic capabilities to adapt to strategies, so as to help them adapt to external environment and improve enterprise performance (Canhoto et al., 2021; Li H., 2022). When the digital transformation strategy of enterprises goes deeper and deeper, as the understanding and recognition degree of the digital transformation strategy of top managers is further deepened, the “behavior convention” which is adapted to the digital transformation strategy is strengthened in the enterprise operation. Enterprises gradually form dynamic capabilities of efficient acquisition, integration and allocation of resources to improve performance (Li H., 2022). Based on the above analysis, this study proposes the following hypothesis.

*Hypothesis 4*: Top managerial cognition positively moderates the relationship between enterprise digital transformation and enterprise performance.

**Figure 1:**
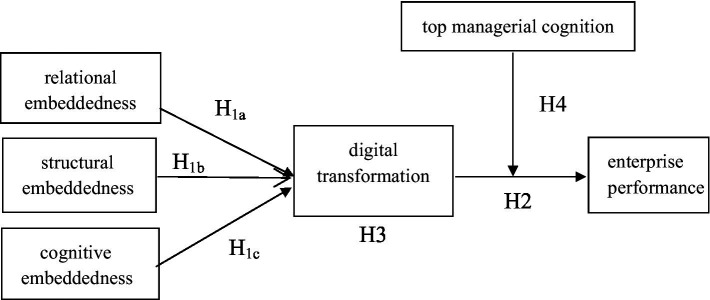
Mediator and moderating effect of the relationship between network embeddedness and enterprise performance.

As can be seen from Figure 1, network embeddedness includes relational embeddedness, structural embeddedness and cognitive embeddedness, which, respectively, represent the relationship intimacy, dominant position and mutual recognition degree between individuals. The higher the degree of relational embeddedness, the more active the digital technology and information sharing behavior between enterprises, and the deeper the cooperation. The higher the degree of structural embeddedness, it means that the enterprise occupies a dominant position in the social network and is more able to obtain high-quality resources and deploy other resources in the social network, which is conducive to the implementation of the digital transformation strategy. A higher degree of cognitive embeddedness means that a common vision is formed between enterprises to promote heterogeneous knowledge such as digital technology to be accepted and understood by other enterprises within the network and to jointly solve digital problems. In conclusion, network embeddedness has a positive impact on enterprises’ implementation of digital transformation. Digital transformation has a positive impact on enterprise performance mainly through reducing enterprise cost and improving capital use efficiency. Top managerial cognition is the basis for shaping the dynamic capability of enterprises. In the implementation process of enterprises’ digital transformation, top managerial cognition can continuously shape the dynamic capability of adapting to the digital transformation strategy and reduce organizational inertia, thus affecting the level of enterprise performance as a moderating variable in the research model.

## Materials and methods

### Setting and sample

The research objects of this study are middle and senior managers of enterprises in Shanghai, Hong Kong, Beijing, Jinan and Qingdao of Shandong Province, Hangzhou, Ningbo and Zhoushan of Zhejiang Province. In order to ensure data quality, research enterprises are selected according to the following three criteria. First of all, The enterprise must be established for more than 3 years, and form certain characteristics in the field of technology research and development. Secondly, Each enterprise must have at least 3 senior managers to fill in the questionnaire. Thirdly, The enterprise is currently in the process of digital transformation or has completed the process of digital transformation. Questionnaires were distributed on site in paper form. A total of 313 questionnaires were collected, and 239 valid questionnaires were obtained after excluding those that did not meet the requirements. The effective rate was 76.4%. In the final analysis of the sample, the characteristics of managers are as follows: male account for 56.8%, female account for 43.2%; 36.6% were under the age of 30, 49.8% were between 31 and 45, and 13.7% were between 46 and 60. The proportion of graduates with college degree or below is 7.5%, bachelor degree 83.7%, master degree 5.7% and doctor degree 3.0%. The organizational characteristics are as follows: the technology-intensive industries (electronic information, biomedicine) account for 46.3%, and the non-technology-intensive industries (food, clothing) account for 53.7%; 10.6% were established for 3–5 years, 68.3% were established for 6–10 years and 21.1% were established for more than 10 years. The number of enterprises with less than 100 employees account for 3.1%, 100–199 employees 50.2%, 200–499 employees 38.3%, 500–999 employees 7.5%, and more than 1,000 employees 0.9%; State-owned enterprises account for 38.8%, private enterprises account for 42.3%, Sino-foreign joint ventures account for 8.8%, and wholly foreign-owned enterprises account for 10.1%.

### Methods

To test the validity of variables, we first used Lisrel 8.7 to conduct confirmatory factor analysis. Second, we use the hierarchical regression analysis to test the effect of independent variables on dependent variables, the effect of independent variables on mediator, and the effect of independent variables on dependent variables after introducing mediator, in accordance with the three steps suggested by Baron and Kenny (1986). Finally, to make the conclusion more robust, mediation effect and moderated mediation effect were both analyzed in accordance with Hayes (2013). According to PROCESS procedures recommended by Preacher and Hayes (2008), bootstrapping with 5,000 replications was employed to derive standard errors, estimates, and bias-corrected 95% confidence intervals.

### Measures

Likert 5-point scale was used to measure each variable (1 = strongly disagree, 5 = strongly agree).

#### Independent variable

The independent variable, network embeddedness is divided into three dimensions: relational embeddedness, structural embeddedness and cognitive embeddedness (Nahapiet and Ghoshal, 1998), and each dimension has corresponding items to measure. The scale developed by Gulant and Sytch was used for relational embeddedness, with a total of 3 items. Cronbach alpha was 0.881. The example item was “I have a high frequency of contact with core stakeholders.” The scale developed by Granovetter was used for structural embeddedness, with a total of 4 items. Cronbach alpha was 0.861. The example item was “I have established more contacts with core stakeholders.” The scale developed by Xiang Sheng and Wei Jiang was used for cognitive embeddedness, with a total of 3 items. Cronbach alpha was 0.835. The example item is “I value the common vision when cooperating and communicating with other enterprises on behalf of the enterprise.”

#### Dependent variable

The dependent variable, enterprise performance, was measured with 3 items. The scale developed by Rhee et al. (2010) was adopted for enterprise performance. Cronbach alpha was 0.875, and the example item is “In the past three years, our company has had a higher market share than its competitors.”

#### Mediator

The mediator, digital transformation, was measured with 5 items. The scale developed by Li (2020) was adopted for digital transformation. Cronbach alpha was 0.886. The example item is “My company comprehensively promotes digital design, manufacturing and management.”

#### Moderator

Top managerial cognition refers to the reactivity measurement index developed by Smith et al. (2010), and this scale is adapted according to the enterprise technology situation. There are 5 items in total. Cronbach alpha is 0.840, and the example item is “I usually judge the investment risk of new technology based on sufficient information.”

#### Control variables

With reference to previous research results, gender (0 = male, 1 = female), age (1 = less than 30 years old, 2 = 31–45 years old, 3 = 46–60 years old) and educational background (1 = junior college or below, 2 = undergraduate, 3 = master, 4 = doctor) are selected as control variables in this study. At the organizational level, this study selects the industry of the enterprise (1 = technology-intensive industry, 2 = non-technology-intensive industry), the years of establishment (1 = 3–5 years, 2 = 6–10 years, 3 = more than 10 years), the size of the enterprise (1 = less than 100 people, 2 = 100–199 people, 3 = 200–499 people, 4 = 500–999 people, 5 = more than 1,000 employees), the nature of the enterprise (1 = state-owned enterprise, 2 = private enterprise, 3 = Sino-foreign joint venture, 4 = wholly foreign-owned) as the control variable.

## Results

Based on the above assumptions and descriptions, we can predict that network embeddedness will certainly have a positive impact on digital transformation, because network embeddedness represents mutual trust, advantage position and mutual recognition degree among interconnected enterprises. Digital transformation has a positive impact on enterprise performance because it will digitally empower business processes and improve customer dialog. Digital transformation plays an intermediary role between network embeddedness and enterprise performance. Top managerial cognition is more conducive to shaping the dynamic capability to adapt to digital transformation and improving enterprise performance.

### Test of validity

First, this study tested the factor loading, CR and AVE of each variable, as shown in Table 1. It is found that the factor loads of relational embeddedness, structural embeddedness, cognitive embeddedness, digital transformation, top managerial cognition and enterprise performance are 0.872–0.921, 0.729–0.896, 0.843–0.917, 0.807–0.871, 0.717–0.860, and 0.865–0.927respectively, all of which are greater than the threshold 0.5. CR are 0.928, 0.913, 0.902, 0.918, 0.887, and 0.925 respectively, all of which are greater than the threshold of 0.8. The AVE are 0.810, 0.725, 0.755, 0.691, 0.612, and 0.803 respectively, all of which are greater than the threshold value 0.5. The above results show that all variables have good convergent validity.

**Table 1:** Test of convergence validity.

Variable	Item of measurement	Factor loading	CR	AVE
Relational embeddedness	I’m in frequent contact with core stakeholders.	0.907	0.928	0.810
	My work with the core stakeholders keeps my promises.	0.921		
	My interactions with core stakeholders are very good.	0.872		
Structural embeddedness	I make more connections with core stakeholders.	0.729	0.913	0.725
	I have strong influence among core stakeholders.	0.896		
	I have expertise that is scarce.	0.890		
	I serve as an important bridge between the core stakeholders.	0.879		
Cognitive embeddedness	I value shared vision when working with others on behalf of my enterprise. Business	0.845	0.902	0.755
	I value common values in cooperation with other enterprises on behalf of my enterprise.	0.843		
	On behalf of enterprises, I attach importance to the consistency of communication methods in communication and cooperation	0.917		
Digital transformation	I work in a company that promotes digital design, manufacturing and management.	0.871	0.918	0.691
	My company develops digital products and services.	0.818		
	I work for a company that is willing to spend its energy promoting and advertising digital skills and management knowledge.	0.837		
	The consensus within my company is that the adoption of digital technology and digital management is beneficial to the development of the enterprise.	0.821		
	I work for a company that uses digital technology to transform and upgrade existing products, services and processes.	0.807		
Top managerial cognition	My current technology is in a highly competitive market.	0.722	0.887	0.612
	I will consider the development opportunities of my partners when making the technical development plan.	0.860		
	I usually judge the investment risk of a new technology based on enough information.	0.747		
	I’m better suited to start a new technology field.	0.853		
	When faced with a new technology problem, I was able to quickly identify the key features of the problem and propose an alternative solution.	0.717		
Enterprise performance	In the past 3 years, our company has achieved higher market share compared with our competitors.	0.927	0.925	0.803
	In the past 3 years, we have enjoyed higher profit margins compared with our competitors.	0.865		
	Over the past 3 years, our company has had a higher revenue growth rate than our competitors.	0.896		

Secondly, Lisrel 8.7 is used to test the discriminative validity of constructs among the six factors involved in this study, and the results are shown in Table 2. The results show that χ^2^/df = 1.982, which is less than the threshold value 3. RMSEA = 0.066, which is less than the threshold 0.08. CFI = 0.96, NFI = 0.92, greater than the threshold of 0.9. The above results are in line with the research criteria, indicating that all variables have good discriminative validity. At the same time, seven alternative models are set up according to conceptual similarity to show that the assumption model setting is reasonable. When relational embeddedness and structural embeddedness were combined into one factor, the results showed that χ^2^/df = 4.569, RMSEA = 0.125, CFI = 0.88, NFI = 0.85, and some indexes were greater than the corresponding threshold. Similarly, when other variables are combined into a factor, not all indicators can meet the threshold requirements. Therefore, this indicates that the six factors in the model assumed in this study have good discriminative validity.

**Table 2:** Discriminative validity test.

Model	χ^2^	df	χ^2^/df	RMSEA	SRMR	CFI	NFI
Six factor model	426.06	215	1.982	0.066	0.060	0.96	0.92
Five factor model 1	1005.17	220	4.569	0.125	0.100	0.88	0.85
Five factor model 2	792.93	220	3.604	0.107	0.130	0.90	0.87
Five factor model 3	759.62	220	3.453	0.104	0.096	0.91	0.87
Four factor model	1317.73	224	5.883	0.146	0.120	0.83	0.80
Three factor model	1648.24	227	7.261	0.166	0.140	0.77	0.75
Two factor model	2246.92	229	9.812	0.197	0.150	0.71	0.69
Single factor model	2417.08	230	10.509	0.204	0.150	0.68	0.65

Five-factor model 1: relational embeddedness + structural embeddedness, cognitive embeddedness, digital transformation, top managerial cognition, enterprise performance; Five-factor model 2: relational embeddedness, structural embeddedness + cognitive embeddedness, digital transformation, top managerial cognition, and enterprise performance; Five factor model 3: relational embeddedness + cognitive embeddedness, structural embeddedness, digital transformation, top managerial cognition, enterprise performance; Four-factor model: relational embeddedness + structural embeddedness + cognitive embeddedness, digital transformation, top managerial cognition, and enterprise performance; Three factor model: relational embeddedness + structural embeddedness + cognitive embeddedness, digital transformation + enterprise performance, top managerial cognition; Two-factor model: relational embeddedness + structural embeddedness + cognitive embeddedness, digital transformation + top managerial cognition + enterprise performance; Single factor model: relational embeddedness + structural embeddedness + cognitive embeddedness + digital transformation + top managerial cognition+ enterprise performance.

### Descriptive statistical analysis

Table 3 lists the mean value, standard deviation and correlation coefficient of each variable. It can be seen that relational embeddedness is positively correlated with digital transformation (*r* = 0.513, *p* < 0.01), top managerial cognition (*r* = 0.316, p < 0.01), and enterprise performance (*r* = 0.218, *p* < 0.01). Structural embeddedness has a significant positive correlation with digital transformation (*r* = 0.197, *p* < 0.01), but has no significant correlation with top managerial cognition (*r* = 0.059, n.s.) and enterprise performance (*r* = 0.034, n.s.). Cognitive embeddedness has positively correlation with digital transformation (*r* = 0.422, *p* < 0.01), top managerial cognition (*r* = 0.265, *p* < 0.01), and enterprise performance (*r* = 0.341, *p* < 0.01). Digital transformation is positively correlated with top managerial cognition (*r* = 0.419, *p* < 0.01) and enterprise performance (*r* = 0.285, *p* < 0.01). There was a significant positive correlation between top managerial cognition and enterprise performance (*r* = 0.439, *p* < 0.01). This sets the stage for hypothesis testing. At the same time, the AVE of the corresponding variable is greater than its correlation coefficient with other variables, which indicates that all variables have good discriminative validity.

**Table 3:** Mean value, standard deviation, and correlation coefficient of each variable.

Variable	M	Sd.	1	2	3	4	5	6
Relational embeddedness	4.317	0.675	(0.900)					
Structural embeddedness	4.406	0.796	0.288^**^	(0.851)				
Cognitive embeddedness	3.632	0.915	0.292^**^	0.169^*^	(0.869)			
Digital transformation	4.007	0.670	0.513^**^	0.197^**^	0.422^**^	(0.831)		
Top managerial cognition	3.547	0.845	0.316^**^	0.059	0.265^**^	0.419^**^	(0.782)	
Enterprise performance	3.364	0.856	0.218^**^	0.034	0.341^**^	0.285^**^	0.439^**^	(0.896)

The diagonal line is AVE; There are too many control variables not listed in this table. ^*^p < 0.05,^**^p < 0.01, the same below.

### Test of hypothesis

In order to test the relationship between the hypotheses, SPSS23.0 was used to conduct hierarchical regression analysis, and the results were shown in Table 4. With the method of Baron and Kenny (1986), this paper first tested the influences of independent variables on mediating variables and dependent variables, then tested the influences of mediating variables on dependent variables, and finally tested the changes in the influences of independent variables on dependent variables after controlling the mediating variables. First, this paper examines the influence of independent variables on mediating variables. Model M2 shows that network embeddedness has a significant positive effect on digital transformation after controlling the characteristic variables of managers and organizations (β = 0.562, *p* < 0.01). **Hypothesis 1 is supported**. M3 showed that both relational embeddedness and cognitive embeddedness had significant positive effects on digital transformation (β = 0.410, *p* < 0.01; β = 0.308, *p* < 0.01), while structural embeddedness had no significant effect on digital transformation (β = 0.038, n.s.). **Hypotheses 1a and 1c are supported,** but hypothesis 1b is not. Secondly, this paper examines the influences of independent variables and mediating variables on dependent variables. Based on M4, the M5 model in this paper incorporates network embeddedness into the equation, and it is found that network embeddedness has a significant positive impact on enterprise performance (β = 0.352, *p* < 0.01). M6 model shows that relational embeddedness and cognitive embeddedness have significant positive effects on enterprise performance (β = 0.243, *p* < 0.01; β = 0.221, *p* < 0.01), while structural embeddedness has no significant effect on enterprise performance (β = 0.006, n.s.). M7 model shows that digital transformation has a significant positive impact on enterprise performance (β = 0.562, *p* < 0.01). **Hypothesis 2 is supported.** On the basis of M6, this paper further examines the mediating effect. Through M8 model, it is found that digital transformation has a significant positive impact on enterprise performance (β = 0.241, *p* < 0.01). The influence of relational embeddedness and cognitive embeddedness on enterprise performance decreased from 0.243 and 0.221 to 0.144 and 0.147, and the significance decreased from 1 to 5%. This indicates that digital transformation plays a partial mediating role between relational embeddedness, cognitive embeddedness and enterprise performance. Similarly, digital transformation has no mediating effect on structural embeddedness. **Hypothesis 3 is partially supported**. In order to test the moderating effect of top managerial cognition on digital transformation and enterprise performance, this paper further introduces the interaction term between top managerial cognition and digital transformation based on M8 and M9. M10 shows that the interaction term has a significant positive impact on enterprise performance (β = 0.141, *p* < 0.05). This indicates that the top managerial cognition has a positive moderating effect on the relationship between digital transformation and enterprise performance. **Hypothesis 4 is supported.**

**Table 4:** Results of hypothesis testing.

Variable	Digital transformation			Enterprise performance						
	M1	M2	M3	M4	M5	M6	M7	M8	M9	M10
Gender	−0.004	0.004	−0.026	0.063	0.068	0.048	0.065	0.054	0.044	0.043
Age	0.054	0.013	−0.017	0.077	0.051	0.031	0.056	0.035	0.021	0.022
Education	−0.012	−0.001	−0.053	−0.090	−0.083	−0.117	−0.085	−0.104	−0.080	−0.079
Nature of the enterprise	−0.009	0.029	0.005	−0.028	−0.004	−0.018	−0.025	−0.019	−0.013	−0.014
Size of enterprise	0.094	0.116	0.130^*^	0.197^**^	0.211^**^	0.220^**^	0.161^*^	0.189^**^	0.175^**^	0.173^**^
Type of enterprise	0.106	0.140^*^	0.084	0.193^**^	0.214^**^	0.178^**^	0.153^*^	0.158^*^	0.126^*^	0.123^*^
Years of establishment	0.114	−0.033	0.038	0.022	−0.069	−0.023	−0.021	−0.032	−0.001	−0.001
Network embeddedness		0.562^**^			0.352^**^					
Relational embeddedness			0.410^**^			0.243^**^		0.144^*^	0.123	0.127
Structural embeddedness			0.038			0.006		−0.004	−0.003	−0.002
Cognitive embeddedness			0.308^**^			0.221^**^		0.147^*^	0.061	0.054
Digital transformation							0.378^**^	0.241^**^	0.210^**^	0.215^**^
Top managerial cognition									0.287^**^	0.274^**^
Transformation × cognition										0.141^*^
*R* ^2^	0.038	0.326	0.377	0.119	0.232	0.255	0.257	0.291	0.358	0.387
VIF_max_	1.898	1.904	1.921	1.898	1.904	1.921	1.901	1.921	1.924	1.926
*F*	1.249	13.170^**^	13.093^**^	4.231^**^	8.212^**^	7.394^**^	9.412^**^	8.028^**^	9.930^**^	9.481^**^

The standardized coefficients are shown in the table.

In addition, with the mean ± standard deviation of top managerial cognition representing high and low value, the impact of digital transformation on enterprise performance under different levels of top managerial cognition is analyzed. Compared with the low level of top managerial cognition, the digital transformation can bring higher enterprise performance under the high level of top managerial cognition.

### Robustness test

In order to make the conclusion more robust and avoid the errors in the first category of statistics, the PROCESS macro confidence interval was used again for bootstrap (sample = 5,000) analysis of the mediation effect, and the results were shown in Table 5. As for the determination of mediating effect, this study adopts the current mainstream view, that is, whether the product term of the regression coefficient from independent variable to intermediary variable and the regression coefficient from intermediary variable to dependent variable is significantly non-zero. The results show that the indirect effect of relational embeddedness on enterprise performance through digital transformation is 0.224, and 95%CI is [0.137, 0.339], excluding zero. The direct effect was 0.172, 95%CI [−0.001, 0.344], including zero. The above data indicate that digital transformation plays a mediating role between relational embeddedness and enterprise performance. Similarly, digital transformation does not play a significant mediating role between structural embeddedness and enterprise performance, but plays a significant mediating role between cognitive embeddedness and enterprise performance.

**Table 5:** Robustness test of mediating effect.

Independent variable	Class of effect	Estimate	SE	BC 95%CI	
				Lower	Upper
Relational embeddedness	Indirect effect	0.224	0.051	0.137	0.339
	Direct effect	0.172	0.088	−0.001	0.344
Structural embeddedness	Indirect effect	0.089	0.037	−0.026	0.173
	Direct effect	−0.026	0.066	−0.156	0.103
Cognitive embeddedness	Indirect effect	0.146	0.034	0.089	0.221
	Direct effect	0.099	0.061	−0.022	0.220

Similar to the mediating effect, in order to have a robust research conclusion on the moderating effect, the bootstrap method was used again in this paper to test the moderating effect of top managerial cognition on the relationship between digital transformation and enterprise performance. As shown in Table 6. In this study, the conditional indirect effects under different values of moderator variable were directly obtained through the PROCESS operation, and the different values were automatically operated to reduce one standard deviation and increase one standard deviation, respectively, on the basis of the mean value of moderator variables, forming low value and high value. The results show that under the higher level of top managerial cognition, the effect of digital transformation on enterprise performance is 0.221, 95%CI is [0.108, 0.359], excluding zero. However, in the situation of low level of top managerial cognition, the effect of digital transformation on enterprise performance is 0.129, 95%CI is [0.027, 0.250], excluding zero. In order to further reveal the differences of the effects in different levels of top managerial cognitive situations, this paper further tested the moderating effect of mediation. The results show that INDEX is 0.053 and 95%CI is [−0.028, 0.146], including zero. The above results show that the top managerial cognition has no significant moderating effect on the relationship between digital transformation and enterprise performance. Similarly, when the independent variable is structural embeddedness, the top managerial cognition has no significant moderating effect on the relationship between digital transformation and enterprise performance. When the independent variable is cognitive embeddedness, the top managerial cognition has a significant positive moderating effect on the relationship between digital transformation and enterprise performance.

**Table 6:** Robustness test of moderating effect.

Independent variable	Conditional indirect effect					Moderating effect with mediator			
	Moderator	Effect	SE	BC 95%CI		INDEX	SE	BC 95%CI	
				Lower	Upper			Lower	Upper
Relational embeddedness	Low	0.129	0.056	0.027	0.250	0.053	0.045	−0.028	0.146
	High	0.221	0.064	0.108	0.359				
Structural embeddedness	Low	0.056	0.029	0.016	0.131	0.015	0.017	−0.010	0.061
	High	0.081	0.040	0.023	0.179				
Cognitive embeddedness	Low	0.103	0.036	0.041	0.180	0.047	0.026	0.023	0.082
	High	0.149	0.042	0.077	0.242				

## Discussion and conclusion

In this study, I find that relational embeddedness and cognitive embeddedness have a positive impact on digital transformation, while structural embeddedness has no significant impact on digital transformation. Through communication and cooperation with other stakeholders, enterprises can obtain information, capital and other resources to promote digital transformation. Moreover, mutual recognition and common vision and goals between enterprises deepen their understanding of digital knowledge, which is conducive to the implementation of digital transformation strategy. Digital transformation improves enterprise performance by reducing production cost and transaction cost. In addition, in the path of the influence of digital transformation on enterprise performance, the top managerial cognition, as a moderating variable, can shape the dynamic capability to adapt to the external environment and has an important impact on the mechanism of the influence of digital transformation on enterprise performance. These results indicate that the close external network connection and the successful shaping of internal dynamic capability play an important role in the influence path of enterprise digital transformation on enterprise performance.

### Theoretical implications

This study contributes to the literature in two ways. First of all, many scholars have studied the impact of digital transformation on enterprises. For example, the positive impact of digital transformation on enterprises (e.g., Dubey et al., 2019; Di and Varriale, 2020), negative effects (e.g., Kohtamaki et al., 2020; Ahmadova et al., 2021) and the U-shaped relationship between the two (Li L. X., 2022). Digital transformation facilitates information sharing among enterprises, optimizes the mode of collaboration, and promotes the product innovation activities of enterprises (Marion and Fixson, 2020). Digital transformation has promoted the transformation of business model and realized the deep integration of digitalization and servitization (Favoretto et al., 2022). Using relationships to look at the relationship between prospective suppliers and customers (Kamalaldin et al., 2020), and redefine the capabilities of service-oriented participants in the context of digital transformation (Marcon et al., 2022). The above literature describes the impact of digital transformation on various aspects of enterprises, but there are few literatures on the driving factors of digital transformation. In fact, corresponding technical knowledge and experience are equally important for enterprises to carry out digital transformation (e.g., Cenamor et al., 2017; Ardolino et al., 2019). In the social network, it is necessary for enterprises to cooperate with other enterprises to make up for the shortage of key resources needed for digital transformation. In view of the lack of research on the predrivers of enterprise digital transformation, based on the embeddedness theory, this study discusses and verifies that enterprises’ strengthening external network cooperation can promote enterprise digital transformation. It provides a more systematic analysis framework for understanding the formation mechanism of enterprise digital transformation performance and enriches the relevant literature of digital transformation.

Secondly, existing literature emphasizes the important role of dynamic capability in enterprise innovation. For example, dynamic capability is conducive to enterprise product development and relearning (Chen et al., 2021), and innovation can be realized through rational resource allocation. As a method of dynamic capability, design thinking can help organizations perceive new opportunities on the basis of understanding user needs, and is regarded as a means to improve organizational innovation capability (e.g., Magistretti et al., 2021a,b). In a word, dynamic capability plays an important role in obtaining and maintaining competitive advantage. However, dynamic capability is different from ordinary capability, which is complicated to establish under the circumstance of limited organization resources. The top managerial cognition will affect the recognition of important information, what strategies should be adopted to adapt to the dynamic environment, and how to use the internal resources of the organization to shape the core competitiveness. Therefore, this study introduces the high-level theory and systematically examines the positive impact of digital transformation on enterprise performance under the context of top managerial cognition from the perspective of dynamic capability shaping. This study affirms the important moderating role of top managerial cognition in the process of digital transformation affecting enterprise performance by shaping dynamic capabilities, opens the “black box” of enterprise performance influencing mechanism under the background of digital transformation, and enriches the existing literature.

### Managerial implications

This study also provides some implications for practitioners. First, Social network provides abundant resources and information for enterprise development, while digital transformation is conducive to strengthen the connection of enterprises and improve the efficiency of resource interaction, so as to promote the improvement of enterprise performance. Therefore, enterprises should actively build an information exchange platform with external network subjects, improve the quality of communication and cooperation, and create a good communication environment for their own development. For example, by creating open innovation communities and developing new business models based on digital platforms, enterprises can establish high-frequency and long-term interactive relationships with external stakeholders, actively play the positive role of digital transformation and improve enterprise performance.

Second, digital transformation is not an overnight task. Top managers must be patient and attach importance to shaping dynamic capabilities (Liang et al., 2018). As pointed out by Warner and Wager (2019), dynamic capability is highly valuable in the digital environment, which is conducive to promoting enterprises to achieve digital transformation and improve performance. Magistretti et al. (2021a) also emphasizes this view. It argues that dynamic capabilities such as different design thinking should be used by managers to help them better understand user needs and develop solutions more effectively by putting users at the center. Therefore, according to the research results of this paper and previous research views, if enterprises want to expand the positive impact of digital transformation on performance, they should attach importance to the important role of top managerial cognition in shaping organizational dynamic capabilities, and choose managers who are inclined to actively transform business models by using digital technologies and promote digital servitization. The above methods can help enterprises quickly perceive and respond to changes in the external environment.

### Future research and limitations

There are the following limitations in this study, which need to be further analyzed and explored in future studies. First of all, this study discusses the logic and mechanism of the influence of digital transformation of general enterprises on enterprise performance. However, every enterprise is faced with both general and specific situations when conducting digital transformation. Based on specific situations, the general digital transformation proposed in this study can be further modified and expanded in future studies. Secondly, in the impact of digital transformation on enterprise performance, the impact of industry characteristics can be further investigated, that is, the difference of the impact of digital transformation on enterprise performance with different industry characteristics. Finally, this study discusses the moderating effect of top managerial cognition. Future studies can further enrich the theoretical framework in this field by examining the moderating effect of other management characteristics.

## Data availability statement

The original contributions presented in the study are included in the article/supplementary material, further inquiries can be directed to the corresponding author.

## Author contributions

YL is responsible for writing the overall article, and GZF is responsible for data collection and processing. All authors listed have made a substantial, direct, and intellectual contribution to the work and approved it for publication.

## Conflict of interest

The authors declare that the research was conducted in the absence of any commercial or financial relationships that could be construed as a potential conflict of interest.

## Publisher’s note

All claims expressed in this article are solely those of the authors and do not necessarily represent those of their affiliated organizations, or those of the publisher, the editors and the reviewers. Any product that may be evaluated in this article, or claim that may be made by its manufacturer, is not guaranteed or endorsed by the publisher.

## References

[ref1] Ahmadova G. Delgado-Marquez B. Pedauga L. E. (2021). The curvilinear relationship between digitalization and firm’s environmental performance. Acad. Manage. 2021:13605. doi: 10.5465/AMBPP.2021.13605abstract

[ref2] Ainunnisa R. (2021). The influence of intellectual capital on the firm’s value with profitability as intervening variable (empirical study on banking subsector companies listed on the Indonesia stock exchange (IDX) of the year 2017-2019). Turcomat 12, 713–722. doi: 10.17762/TURCOMAT.V12I4.555

[ref3] Ardolino M. Rapaccini M. Saccani N. Gaiardelli P. Crespi G. Ruggeri C. (2019). The role of digital technologies for the service transformation of industrial companies. Int. J. Prod. Res. 56, 2116–2132. doi: 10.1080/00207543.2017.1324224

[ref01] Baron R. M. Kenny D. A. (1986). The moderator-mediator variable distinction in social psychological research: Conceptual, strategic, and statistical considerations. J. Pers. Soc. Psychol. 51, 1173–1182. doi: 10.1037/0022-3514.51.6.1173 3806354

[ref4] Bavelas A. (1950). Communication patterns in task-oriented groups. J. Acoust. Soc. Am. 22, 725–730. doi: 10.1121/1.1906679

[ref5] Borkar V. R. Carey M. J. Li C. (2012). Big data platforms: what's next? XRDS: crossroads. ACM Magaz. Stud. 19, 44–49. doi: 10.1145/2331042.2331057

[ref6] Canhoto A. I. Quinton S. Pera R. Molinillo S. Simkin L. (2021). Digital strategy aligning in SMEs: a dynamic capabilities perspective. J. Stragegic Inf. Syst. 30:101682. doi: 10.1016/j.jsis.2021.101682

[ref7] Carley K. Palmouist M. (1992). Extracting, representing, and analyzing mental models. Soc. Forces 70, 601–636. doi: 10.2307/2579746

[ref8] Cenamor J. Sjodin D. R. Parida V. (2017). Adopting a platform approach in servitization: leveraging the value of digitalization. Int. J. Prod. Econ. 192, 54–65. doi: 10.1016/j.ijpe.2016.12.033

[ref9] Chen Y. J. Nicole C. Chatura R. (2021). When change is all around: how dynamic network capability and generative NPD learning shape a firm’s capacity for major innovation. J. Prod. Innovat. Manag. 38, 574–599. doi: 10.1111/jpim.12595

[ref10] Cohn B. S. Marriott M. K. (1958). Networks and centres of integration in Indian civilization. J. Soc. Res. 74, 458–459. doi: 10.2307/3557787

[ref11] Corbett C. J. (2018). How sustainable is big data? Prod. Oper. Manag. 27, 1685–1695. doi: 10.1111/poms.12837

[ref12] Cummings J. N. (2004). Work groups, structural diversity, and knowledge sharing in a gobal organization. Manag. Sci. 50, 352–364. doi: 10.1287/mnsc.1030.0134

[ref13] Di V. A. Varriale L. (2020). Blockchain technology in supply chain management for sustainable performance: evidence from the airport industry. Int. J. Inform. Manage. 52, 102014–102304. doi: 10.1016/j.ijinfomgt.2019.09.010

[ref14] Dubey R. Gunasekaran A. Childe S. J. Blome C. Papadopoulos T. (2019). Big data and predictive analytics and manufacturing performance: integrating institutional theory, resource-based view and big data culture. Brit. J. Manage. 30, 341–361. doi: 10.1111/1467-8551.12355

[ref15] Dutton J. E. Jackson S. E. (1987). Categorizing strategic issues: links to organizational action. Acad. Manage. Rev. 12, 76–90. doi: 10.2307/257995

[ref16] Favoretto C. Mendes G. H. S. Oliveira M. G. Cauchick-Miguel P. A. Coreynen W. (2022). From servitization to digital servitization: how digitalization transforms companies' transition towards services. Ind. Market. Manag. 102, 104–121. doi: 10.1016/j.indmarman.2022.01.003

[ref17] Fung M. K. (2006). R & D, knowledge spillovers and stock volatility. Account. Financ. 46, 107–124. doi: 10.1111/j.1467-629X.2006.00166.x

[ref18] Gavetti G. Levinthal D. (2000). Looking forward and looking backward: cognitive and experiential search. Adm. Sci. Q. 45, 113–137. doi: 10.2307/2666981

[ref19] Goldfarb A. Tucker C. (2019). Digital economics. J. Econ. Lit. 57, 3–43. doi: 10.1257/jel.20171452

[ref20] Gonzalez-Brambila C. N. Veloso F. M. Krackhardt D. (2013). The impact of network embeddedness on research output. Policy. Res. 42, 1555–1567. doi: 10.1016/j.respol.2013.07.008

[ref06] Hayes A. F. (2013). Introduction to mediation, moderation, and conditional process analysis. New York, NY: Guilford Press.

[ref21] Kamalaldin A. Linde L. Sjodin D. Parida V. (2020). Transforming provider-customer relationships in digital servitization: a relational view on digitalization. Ind. Market. Manag. 89, 306–325. doi: 10.1016/j.indmarman.2020.02.004

[ref22] Kohtamaki M. Parida V. Patel P. C. Gebauer H. (2020). The relationship between digitalization and servitization: the role of servitization in capturing the financial potential of digitalization. technol. Forecast. Soc. 151:119804. doi: 10.1016/j.techfore.2019.119804

[ref07] Li F. (2020). The digital transformation of business models in the creative industries: A holistic framework and emerging trends. Technovation 92. doi: 10.1016/j.technovation.2017.12.004

[ref23] Li H. (2022). Green innovation, green dynamic capability, and enterprise performance: evidence from heavy polluting manufacturing enterprises in China. Complexity 2022, 1–13. doi: 10.1155/2022/7755964

[ref24] Li L. X. (2022). Digital transformation and sustainable performance: the moderating role of market turbulence. Industr. Market. Manag. 104, 28–37. doi: 10.1016/j.indmarman.2022.04.007

[ref25] Liang L. Fang S. Wei Z. Mao J. Y. (2018). Digital transformation by SME entrepreneurs: a capability perspective. Australas. J. Inf. Syst. 28, 1129–1157. doi: 10.1111/isj.12153

[ref26] Liang S. Li T. (2022). Can digital transformation promote innovation performance in manufacturing enterprises? The mediating role of R&D capability. Sustainability 14:10939. doi: 10.3390/su141710939

[ref27] Lieberman M. B. Asaba S. (2006). Why do firms imitate each other? Acad. Manage. Rev. 31, 366–385. doi: 10.5465/amr.2006.20208686

[ref28] Magistretti S. Ardito L. Petruzzelli A. M. (2021a). Framing the microfoundations of design thinking as a dynamic capability for innovation: reconciling theory and practice. J. Prod. Innovat. Manag. 38, 645–667. doi: 10.1111/jpim.12586

[ref29] Magistretti S. Pham C. T. A. Dell'Era C. (2021b). Enlightening the dynamic capabilities of design thinking in fostering digital transformation. Ind. Market. Manag. 97, 59–70. doi: 10.1016/j.indmarman.2021.06.014

[ref30] Marcon E. Marcon A. Ayala N. F. Frank A. G. Story V. Burton J.. (2022). Capabilities supporting digital servitization: a multi-actor perspective. Ind. Market. Manag. 103, 97–116. doi: 10.1016/j.indmarman.2022.03.003

[ref31] Marion T. J. Fixson S. K. (2020). The transformation of the innovation process: how digital tools are changing work, collaboration, and organizations in new product development. J. Prod. Innovat. Manag. 38, 192–215. doi: 10.1111/jpim.12547

[ref32] Mikalef P. Boura M. Lekakos G. Krogstie J. (2018). Big data analytics capabilities and innovation: the mediating role of dynamic capabilities and moderating effect of the environment. Brit. J. Manage. 30, 272–298. doi: 10.1111/1467-8551.12343

[ref33] Mikalef P. Van de Wetering R. Krogstie J. (2021). Building dynamic capabilities by leveraging big data analytics: the role of organizational inertia. Inf. Manag. 58:103412. doi: 10.1016/j.im.2020.103412

[ref34] Moeuf A. Pellerin R. Lamouri S. Tamayo-Giraldo S. Barbaray R. (2018). The industrial management of SMEs in the era of industry 4.0. Int. J. Prod. Res. 56, 1118–1136. doi: 10.1080/00207543.2017.1372647

[ref35] Nadkarni S. Narayanan V. K. (2007). Strategic schemas, strategic flexibility, and firm performance: the moderating role of industry clockspeed. Strategic. Manage. J. 28, 243–270. doi: 10.1002/smj.576

[ref36] Nahapiet J. Ghoshal S. (1998). Social capital, intellectual capital and the organization advantage. Acad. Manage. Rev. 23, 242–266. doi: 10.5465/amr.1998.533225

[ref37] Papadopoulos T. Singh S. P. Spanaki K. Gunasekaran A. Dubey R. (2022). Towards the next generation of manufacturing: implications of big data and digitalization in the context of industry 4.0. Prod. Plan. Control 33, 101–104. doi: 10.1080/09537287.2020.1810767

[ref38] Pomegbe W. W. K. Li W. Dogbe C. S. K. Otoo C. O. A. (2020). Enhancing the innovation performance of small and medium-sized enterprises through network embeddedness. J. Competitiveness. 12, 156–171. doi: 10.7441/joc.2020.03.09

[ref02] Preacher K. J. Hayes A. F. (2008). Asymptotic and resampling strategies for assessing and comparing indirect effects in multiple mediator models. Behav. Res. Methods 40, 879–891. doi: 10.3758/BRM.40.3.879 18697684

[ref03] Rhee J. Park T. Lee D. H. (2010). Drivers of innovativeness and performance for innovative SMEs in South Korea: Mediation of learning orientation. Technovation. 30, 65–75. doi: 10.1016/j.technovation.2009.04.008

[ref39] Sankaranarayanan R. Sundararajan A. (2010). Electronic markets, search costs, and firm boundaries. Inform. Syst. Res. 21, 154–169. doi: 10.1287/isre.1090.0235

[ref04] Smith W. K. Binns A. Tushman M. L. (2010). Complex business models: managing strategic paradoxes simultaneously. Long. Range. Plann. 43, 448–461. doi: 10.1016/j.lrp.2009.12.003

[ref40] Tiwana A. (2008). Do bridging ties complement strong ties? An empirical examination of alliance ambidexterity. Strategic. Manage. J. 29, 251–272. doi: 10.1002/smj.666

[ref05] Trenerry B. Chng S. Wang Y. Suhaila Z. S. Lim S. S. Lu H. Y.. (2021). Preparing workplaces for digital transformation: an integrative review and framework of multi-level factors. Front. Psychol. 12, 62–65. doi: 10.3389/fpsyg.2021.620766 PMC8021873 33833714

[ref41] Tsou H. T. Chen J. S. (2021). How does digital technology usage benefit firm performance? Digital transformation strategy and organizational innovation as mediators. Technol. Anal. Strateg. 1-14, 1–14. doi: 10.1080/09537325.2021.1991575

[ref42] Wang H. Cao W. Wang K. (2022). F. Digital transformation and manufacturing firm performance: evidence from China. Sustainability 14:10212. doi: 10.3390/su141610212

[ref43] Warner S. R. Wager M. (2019). Building dynamic capabilities for digital transformation: an ongoing process of strategic renewal. Long Range Plann. 52, 326–349. doi: 10.1016/j.lrp.2018.12.001

[ref44] Xie M. H. Zhao Y. Y. Liu Y. Y. (2021). A research on the influence path of network ties, resource acquisition and organizational learning interaction on strategic performance—a longitudinal case study based on the Great Wall motor company. Sci. Res. Manage. 42, 57–69. doi: 10.19571/j.cnki.1000-2995.2021.05.007

[ref45] Zhang Y. Li Y. Li H. Y. (2014). FDI spillovers over time in an emerging market: the roles of entry tenure and barriers to imitation. Acad. Manage. J. 57, 698–722. doi: 10.5465/amj.2011.0351

[ref46] Zhang T. Shi Z. Z. Shi Y. R. Chen N. J. (2022). Enterprise digital transformation and production efficiency: mechanism analysis and empirical research. Econ. Res-Ekon. Istraz. 35, 2781–2792. doi: 10.1080/1331677X.2021.1980731

